# Radiotherapy of oligometastatic prostate cancer: a systematic review

**DOI:** 10.1186/s13014-021-01776-8

**Published:** 2021-03-09

**Authors:** Paul Rogowski, Mack Roach, Nina-Sophie Schmidt-Hegemann, Christian Trapp, Rieke von Bestenbostel, Run Shi, Alexander Buchner, Christian Stief, Claus Belka, Minglun Li

**Affiliations:** 1 Department of Radiation Oncology, University Hospital, LMU Munich, Marchioninistr. 15, 81377 Munich, Germany; 2 Department of Radiation Oncology, UCSF Helen Diller Family Comprehensive Cancer Center, 1600 Divisadero Street, Suite H 1031, San Francisco, CA 94143-1708 USA; 3 Department of Urology, University Hospital, LMU Munich, Marchioninistr. 15, 81377 Munich, Germany; 4 German Cancer Consortium (DKTK), Munich, Germany

**Keywords:** Oligometastatic prostate cancer, Metastasis‐directed therapy, Radiotherapy, SBRT, ENRT

## Abstract

**Background:**

Due to improved imaging sensitivity, the term “oligometastatic” prostate cancer disease is diagnosed more often, leading to an increasing interest in metastasis-directed therapy (MDT). There are two types of radiation based MDT applied when treating oligometastatic disease: (1) stereotactic body radiation therapy (SBRT) generally used for bone metastases; or (2) SBRT for isolated nodal oligometastases combined with prophylactic elective nodal radiotherapy. This review aims to summarize current evidence data, which may shed light on the optimal management of this heterogeneous group of patients.

**Methods:**

A systematic review of the Medline database through PubMed was performed according to PRISMA guidelines. All relevant studies published up to November 2020 were identified and screened. Fifty-six titles were included. Besides outcome parameters, different prognostic and predictive factors were assessed, including site of metastases, time between primary treatment and MDT, use of systemic therapies, hormone sensitivity, as well as pattern of recurrence.

**Findings:**

Evidence consists largely of retrospective case series and no consistent precise definition of oligometastasis exists, however, most investigators seem to acknowledge the need to distinguish between patients presenting with what is frequently called “synchronous” versus “metachronous” oligometastatic disease. Available data on radiotherapy as MDT demonstrate high local control rates and a small but relevant proportion of patients without progressive disease after 2 years. This holds true for both hormone sensitive and castration resistant prostate cancer diseases. The use of ^68^Ga-PSMA PET/CT for staging increased dramatically. Radiation doses and field sizes varied considerably among the studies. The search for relevant prognostic and predictive factors is ongoing.

**Conclusions:**

To our best knowledge this review on oligometastatic prostate cancer included the largest number of original articles. It demonstrates the therapeutic potential and challenges of MDT for oligometastatic prostate cancer. Prospective studies are under way and will provide further high-level evidence.

## Background

Prostate cancer (PC) is the second most common cancer in men worldwide [1]. After primary treatment with radical prostatectomy or radiation therapy (RT), a relevant proportion of patients develop metastases. Immediate or delayed androgen deprivation therapy (ADT), chemotherapy, chemohormonal therapy and palliative radiotherapy have traditionally been the mainstay of the management of metastatic prostate cancer (MPC) [2].

However, sensitive PSA detection and improved imaging are increasingly leading to the diagnosis of “oligometastatic disease”, which in turn has raised new questions concerning the value of metastasis-directed therapy (MDT) on progression free survival (PFS) and overall survival (OS). The definition of oligometastatic disease is inconsistent and varies from as few as one but up to between three and five metastases. Malignant cells in this state are supposed to have a limited metastatic capacity, accompanied with less aggressive behavior [3]. Accumulating evidence suggests that local MDT could defer disease progression, delay the need of systemic therapies and spare their toxicities. However, in some cases, clinical oligometastasis is only the tip of the iceberg for a subclinical polymetastatic disease. Proper patient selection, as well as the definitions use and relevant endpoints may be critically important to optimal approach oligometastatic disease [4].

**Fig. 1:**
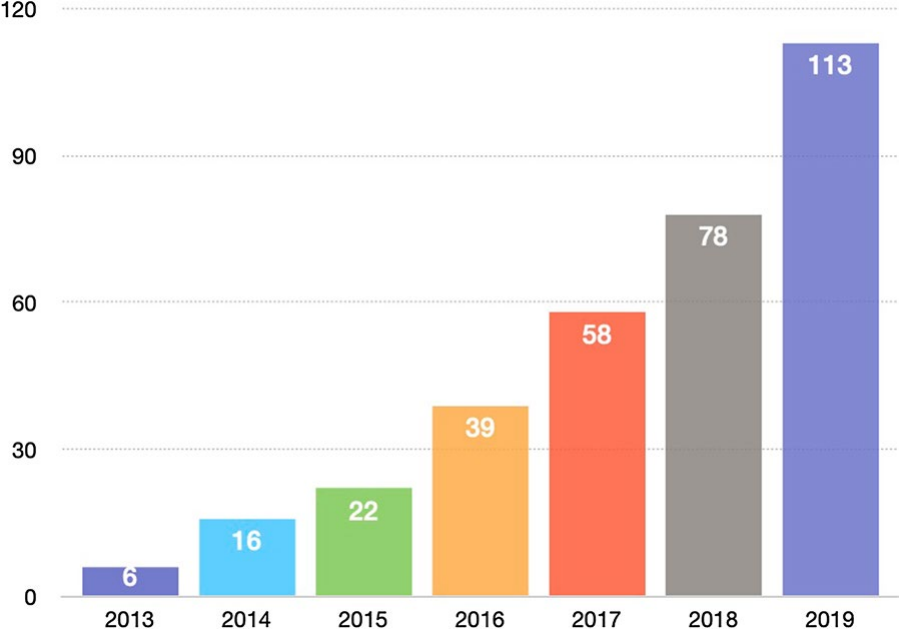
Publications on oligometastatic prostate cancer

Radiotherapy and in particular stereotactic body radiation therapy (SBRT), also sometimes called stereotactic ablative radiotherapy (SABR), presents a logical option for MDT and has been used in many retrospective case series. Figure 1 shows the growing number of publications on oligometastatic PC in the last 7 years.

Timing of the diagnosis of oligometastatic disease seems to be widely held to be important. For example, 68% of expert participants in the advanced prostate cancer consensus conference (APCCC) considered it important to distinguish between patients presenting with what is frequently called “synchronous” versus “metachronous” (appeared later in the course of the disease) oligometastatic disease. Further, despite the lack of high-level evidence, 64% of APCCC members voted for an ablative MDT in metachronous oligometastatic PC [5]. This systematic review provides an overview of the evidence to date for MDT in oligometastatic PC.

## Methods

**Fig. 2:**
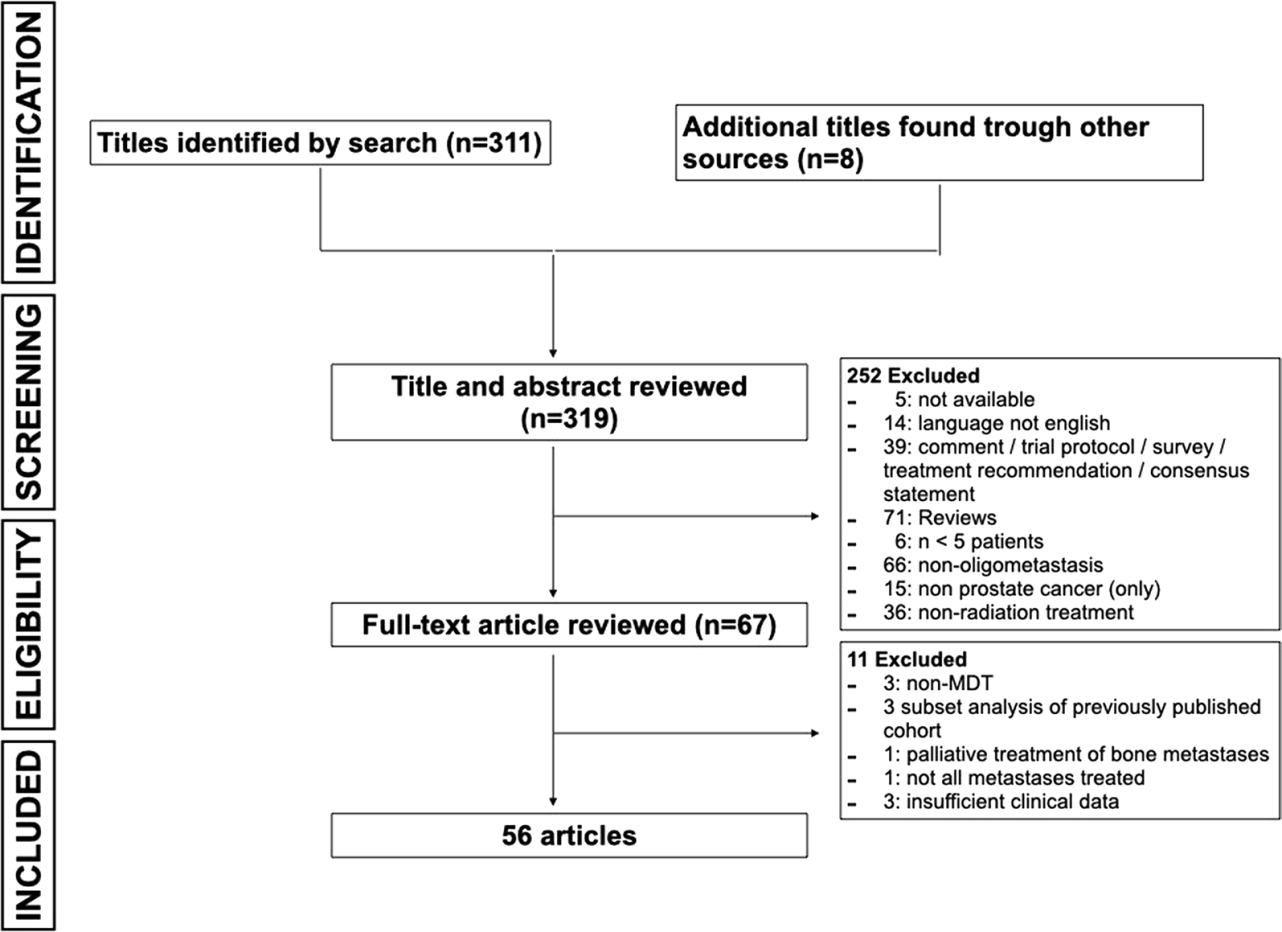
Selection process

A systematic review of the Medline database trough PubMed was performed in October 2019 and updated in November 2020 according to PRISMA (Preferred Reporting Items for Systematic Reviews and Meta-analysis) guidelines. Search terms used were: “prostate cancer”, “radiotherapy”, ”oligometastatic“ and ”metastasis-directed“ or combinations of these. Further inclusion criteria were (a) original article; (b) article in English; (c) accessibility to the full article; (d) cohort consists of oligometastatic PC patients only; (e) MDT was radiotherapy. Additional references were identified from the bibliographies of candidate articles. To minimize publication and reporting bias, case series that comprised fewer than five cases were excluded. Moreover, studies in which not all metastases were treated or just a palliative radiotherapy was conducted were excluded as well. Two studies without sufficient clinical survival data were also excluded. The study selection process is shown in Fig. 2.

## Results

### Oligometastatic prostate cancer and outcome of MDT

Overall, 56 Studies from 2012 to 2020 were included. Study methods and designs are listed in Table 1. The vast majority of the studies were retrospective case series with median follow-up times between 6 and 70 months. Oligometastasis was inconsistently defined, with three and five metastases as the mostly used cut-off value. The inconsistent definition between the studies reflects the ongoing debate and suggests the difficulty of capturing the oligometastatic state by the sheer number of metastases alone. Of note, even though in most studies a maximum of five metastases was used in the inclusion criteria, the majority of patients had one or two metastases.

**Table 1:** Overview of publications included in the systematic review reporting metastasis-directed radiotherapy for oligometastatic prostate cancer

Source	Year	Study design	Maximal numbers of metastases	n (patients/lesions)	Median FU (month)	Imaging method	Site of metastases	Median dose (range) and fractions	Concomitant ADT (%)	Median ADT-FS (month)	Local control	PFS
Jereczek-Fossa [82]	2012	Retrospective analysis	1	19/19	16.9	CT, Bone Scan, Choline-PET/CT	LN 95%, Bone 5%	LN 33 Gy/3 fr, Bone 36 Gy/3 fr	74%	NR	100% (at last FU, median 16.9 mo)	42.6% (30-month PFS)
Tabata [30]	2012	Retrospective analysis	5	35/NR	36	NR	Bone 100%	40 Gy (30–50 Gy)/10–25 fr	100%	–	NR	NR
Ahmed [61]	2013	Retrospective analysis	5	17/21	6	CT, MRI, Choline-PET/CT	LN 5%, bone 90%, viscera 5%	LN 50 Gy/5 fr, Bone: 20 Gy (18–30 Gy)/1–3 fr, Liver 60 Gy/3 fr	88%	NR	100% (6 mo)	74/40% (1-/2-year FFDP)
Berkovic [14]	2013	Retrospective analysis	3	24/29	24	MRI, Bone Scan, FDG-PET/CT, Choline-PET/CT	LN 45%, bone 55%	50 Gy (40–50 Gy)/10 fr (8–10)	0%	38	100% (2 year)	72/42% (1-/2-year CPFS)
Muacevic [31]	2013	Retrospective analysis	2	40/64	10	Choline-PET/CT	Bone 100%	20 Gy (16.5–22 Gy)/1 fr	48%	NR	95.5% (2 year)	NR
Schick [6]	2013	Retrospective analysis	4	50/79	31	MRI, Bone Scan, Choline-PET/CT	LN 63.5%, bone 31.5%, viscera 5%	pelvic LN: 50.4 Gy + Boost to median 65 Gy (44–74 Gy) Bone 28–36 Gy/5–6 fr	98%	NR	NR	58.6/54.5% (3-year CFFS/BPFS)
Decaestecker [15]	2014	Prospective analysis	3	50/70	24	MRI, Choline-PET/CT, FDG-PET/CT	LN 54%, bone 44%, viscera 2%	50 Gy/10 fr, or 30 Gy/3 fr	70%	25	100% (2 year)	64/35% (1-/2-year)
Detti [83]	2015	Retrospective analysis	2	30/39	12	CT, MRI, Choline-PET/CT	LN 100%	Most commonly used 30 Gy (24–36 Gy)/3 fr (1–5)	47%	NR	100% (1 year)	NR
Ponti [57]	2015	Retrospective analysis	2	16/18	29	Choline-PET/CT	LN 100%	35 Gy (12–35 Gy)/5 fr (1–5)	63%	Mean 23.7	94% (6 mo)	45% (2-year BPFS)
Muldermans [7]	2016	Retrospective analysis	5	66/81	16	CT, MRI, Bone Scan, Choline-PET/CT	LN 7.4%, bone 91%, viscera 1%	16 Gy (16–50 Gy)/1 fr (1–5)	64%	NR	82% (2 year)	45/54% (2-year DPFS/BPFS)
Napieralska [75]	2016	Retrospective analysis	4	18/31	16	CT, MRI, Choline-PET/CT	LN 100%	30 Gy (24–45 Gy), 2–5 fr	100%	–	93/70% (1/2 year)	NR
Ost [16]	2016	Retrospective analysis	3	119/163	36	MRI, Choline-PET/CT, FDG-PET/CT	LN 60%, bone 36%, mixed 2%, viscera 1%	≥ 80 Gy BED (α/β 3), SD ≥ 5 Gy	50%	Median time from first SBRT to palliative ADT: 28 m	93/92% (3/5 year)	31/15% (3-/5-year DPFS)
Pasqualetti [17]	2016	Prospective clinical trial	3	29/45	12	Choline-PET/CT	LN 56%, bone 44%	24 Gy/1 fr or 27 Gy/3 fr	0%	39.7	100% (at last FU, median 12 mo)	NR
Wu [8]	2016	Retrospective analysis	3	30/53	33	Bone Scan, Choline-PET/CT	Bone 100%	Short-Course: 20-30 Gy/5–10 fr, Long-course 37.5–50 Gy/15–25 fr	100%	–	75% (3 year)	22.8% (3-year)
Bouman-Wammes [46]	2017	Retrospective double arm	4	43/55	31	Choline-PET/CT	LN (77%), bone (21%), mixed (2)	Most commonly used 30 Gy/3 fr and 35 Gy/5 fr	0%	15.6 m	100% (at last FU, median 2.6 year)	NR
Fodor [26]	2017	Retrospective analysis	2	81/NR	36	Choline-PET/CT	LN (100%)	51.8 Gy/28 fr + SIB 65.5 Gy (TD 50–65 Gy/25–30 fr)	72%	NR	89.8% (3 year)	61.8/42.4%(3-year CRFS/BPFS)
Habl [32]	2017	Retrospective analysis	2	15/20	23	MRI, PSMA-PET/CT, Choline-PET/CT	Bone 100%	25–40 Gy	20%	9.3	100% (2 year)	NR
Ingrosso [71]	2017	Retrospective analysis	2	40/47	24	Choline-PET/CT	LN 100%	Most commonly used: 35 Gy/5 fr and 40 Gy/8 fr (12–50 Gy/1–5 fr)	48%	Mean 26	98% (30 mo)	44% (2-year BPFS)
Jereczek-Fossa [9]	2017	Retrospective analysis	5	94/124	19	CT, MRI, Choline-PET/CT	LN 100%	Most commonly used: 24 Gy (15–36 Gy)/3 fr ( 3–6)	36%	7	84% (2 year)	30% (2-year)
Triggiani [18]	2017	Retrospective double arm	3	141/209	OR: 20.4/OP: 23.4	CT, Bone Scan, Choline-PET/CT	LN 84.1.%/70%, bone 15.8%/30%	116 Gy BED (80–216.6 Gy) (α/β 3 Gy)	24%	OR: 21/OP: 22 (second systemic therapy-free survival)	OR: 92.8%/OP: 90.2% (2 year)	OR: 64.4/43/26.6% (1-/2-/3-year) OP: 43.2/21.6/11.9%
Baumann [45]	2018	Retrospective analysis	5	5/18	11	PSMA-PET/CT	LN 21%, bone 79%	35 Gy/5 fr	0%	NR	88% (at last FU, median 11 mo, determined by PET-response)	NR
Fanetti [33]	2018	Retrospective analysis	5	55/77	25	MRT, PSMA-PET/CT, Choline-PET/CT	Bone 100%	24 Gy (15-30 Gy)/3 fr (1–5)	55%	NR	83/76% (1/2 year)	55/27% (1-/2-year CPFS), 51/13% (1-/2-year BPFS)
Franzese [19]	2018	Retrospective analysis	3	64/90	15.2	Bone Scan, Choline-PET/CT	LN 78%, bone 16%, mixed 3%, viscera 3%	42 Gy (18–60)/2–8 fr	42%	NR	88/84% (1/1.5 year)	52/37% (1-/1.5-year DPFS), 38/25% (1-/1.5-year CPFS)
Guler [50]	2018	Retrospective analysis	3	23/38	7	PSMA-PET/CT	LN 43%, bone 43%, both 23%	45 Gy (30–64 Gy)/15 fr (12–27)	100%	–	100% (1 year)	51% (1 year)
Lépinoy [59]	2018	Retrospective double arm	4	62/88	42	Choline-PET/CT	LN 100%	Extended field: 45–59 Gy in 1.8–2.2 Gy + Boost to median 66 Gy Involved field: 36 Gy (30–66) in 7.5 Gy (2–15)	24%	NR	NR	Extended field: 88.3%/involved field: 55.3% (3-year FFR)
Oertel [21]	2018	Retrospective analysis	LN 5/Bone 1	27/37	NR	CT, MRI, PSMSA-PET/CT, Choline-PET/CT	LN 79%, bone 30%	LN: 63 Gy (30.6–70.2 Gy), Bone: 54 Gy (30–66.6 Gy)	74%	NR	100% (2 year)	LN: 75.4/58.7% (1-/2-year MFS), Bone: 100/83.3%
Ost [60]	2018	Prospective randomized phased II study	3	Surveillance: 31/65, MDT 31/51	36	Choline-PET/CT	LN 55%, bone 39%, mixed 5%, viscera 2%	30 Gy/3 fr	0%	21 versus 13 (*p* = 0.11)	100 vs. 77% at last FU, median 36 mo	NR
Siva [62]	2018	Prospective clinical trial	3	33/50	24	CT, Bone Scan, NaF-PET/CT	LN 36%, bone 61%, mixed 3%	20 Gy/1 fr	33%	2 year ADT-FS: 48%	97/93% (1/2 year)	58/39% (1-/2-year DPFS)
Steuber [84]	2018	Retrospective matched pair analysis	5	263/NR	70	Choline-PET/CT	LN 100%	≥ 80 Gy BED (α/β 3), SD ≥ 5 Gy	NR	NR	NR	NR
Tran [76]	2018	Retrospective analysis	5	53/108	44	MRI, Bone scan, Choline-PET/CT,	LN 100%	45–50.4 Gy on involved LN regions, Boost median to 64.4 (54–69)	100%	–	96.3% (at last FU, median 44 mo)	58.2/43%(5-year DPFS/BPFS)
Henkenberens [51]	2018	Retrospective analysis	2	29/NR	8	PSMA-PET/CT	LN 58.6%, Bone (20.7%), mixed 3.4%	LN (50.4–54.0 Gy), Bone (40 Gy à 2.5 Gy 4x/week)	28%	NR	100% (at last FU, median 8 mo)	75% (1-year DMFS and BPFS)
Loi [85]	2018	Retrospective analysis	2	23/27	22	Choline-PET/CT	LN 100%	24 Gy/1 fr	0%	NR	NR	65/40/26% (6-mo/1-year /2-year BPFS)
Soldatov [54]	2018	Retrospective analysis	5	108/284	18	PSMA-PET/CT	LN 59%, bone 39.6%, viscera 1.4%	LN 50.4–60 Gy in 1.8 Gy, Bone 40 Gy in 2.5 Gy 4x/week	NR	NR	96,3% (at last FU, median 18 mo)	56,5% (BPFS at 18 mo)
Kneebone [20]	2018	Prospective clinical trial	3	57/73	16	PSMA-PET/CT	LN 65%, bone 31%, mixed 4%	LN 30 Gy/3 fr or 50 Gy/5 fr, Bone 20 Gy/1 fr or 24 Gy/2 fr	0%	NR	100% (at last FU, median 16 mo)	46/16% (1-/2-year BPFS)
Cysouw [10]	2018	Retrospective analysis	4	40/50	33	Choline-PET/CT	LN 74%, bone 26%	35 Gy/5 fr or 30 Gy/3 fr	NR	NR	NR	NR
Patel [34]	2019	Retrospective analysis	3	51/64	23	MRI, Bone Scan PSMA-PET/CT, Choline-PET/CT	Bone 100%	30 Gy (24–30 Gy)/3–6 Gy SD	79%	NR	98/95/90% (1/2/3 year)	45/38% (1-/2-year PFS)
Triggiani [24]	2019	Retrospective analysis	5	86/117	31	Bone Scan, Choline-PET/CT	LN 67%, bone 33%	LN 36 Gy/6 fr or 45 Gy/6 fr; Bone 24 Gy/3 fr	0%	Median systemic treatment-free survival 21.8	80% (at last FU, median 31 mo)	52/34% (1-/2-year DPFS)
De Bleser [48]	2019	Retrospective analysis	5	506/764	36	CT, MRI, PSMA-PET/CT, Choline-PET/CT, FDG-PET/CT	LN 100%	SBRT minimal SD 5 Gy, maximum of 10 fractions; ENRT: minimal TD 45 Gy/up to 25 fx	SBRT: 23%, ENRT: 60%	NR	NR	68/77% (SBRT vs. ENRT 3-year MFS, *p* = 0.01)
Bowden [22]	2019	Prospective clinical trial	5	199/NR	35	MRI, Bone Scan, PSMA-PET/CT, Choline-PET/CT	LN 63.3%, bone 22.6%, mixed 12.1%, viscera 2%	50 Gy/10 fr	7%	Median treatment escalation-free survival: 27.1	NR	NR
Gomez-Iturriaga [63]	2019	Prospective clinical trial	5	49/102	24	MRI, Choline-PET/CT	LN 69.4%, bone 26.5%, mixed 4.1%	Bone 24–30 Gy/3 fr, LK 45 Gy + SIB 58 Gy/25 fr or 24 -30 Gy/3 fr	Yes, proportion NR	NR	91.8% (at last FU, median 24 mo)	71% at last FU, 2-year BPFS: 74.2%
Artigas [42]	2019	Retrospective analysis	3	20/30	15	PSMA-PET/CT	LN 60%, bone 30%, viscera 10%	30 Gy/3 fr or Pelvic + SIB up to 66 Gy	0%	2 year ADT-FS: 74%	NR	79/53% (1-/2-year BPFS)
Franzese [49]	2019	Retrospective analysis	5	92/119	22	PSMA-PET/CT, Choline-PET/CT	LN 76%, bone 24%, viszera 3%	42 Gy (18–60 Gy)/2–8 fr	NR	NR	90.9/85.5% (1/3 year)	51.9/20.9% (1-/3-year DPFS), 42.8/16.7% (1-/3-year PFS)
Ong [23]	2019	Retrospective analysis	3	20/26	16	PSMA-PET/CT	LN 75%, bone 15%, mixed 10%	Bone 30 Gy/3 fr, LK 35–40 Gy/5 fr	0%	1-year ADT-FS: 70%	93% (1 year)	62% (1-year)
Nicosia [53]	2019	Retrospective analysis	5	109/155	16	PSMA-PET/CT, Choline-PET/CT	LN 100%	36 Gy (25–48 Gy)/4–7 fr	46%	15	93/87% (1/2 year)	55/33% (1-/2-year PFS), 64/52% (1-/2-year nodal PFS)
Berghen [47]	2019	Retrospective analysis	3	30/45	18	CT, MRI, PSMA-PET/CT, Choline-PET/CT	LN 17%, Bone 66%, both 3%, viscera 3%,	SBRT: EQD2 78–142.8 Gy/3–5 fr, fractionated: 39 Gy/13 fr	NR	Median NEST-FS: 16 mo	NR	median PFS 10 mo
Deantoni [35]	2020	Retrospective analysis	2	39/51	47	CT, Bone Scan, Cholin-PET/CT	Bone 100%	44 Gy	100%	–	95% (at last FU, median 43 mo)	65.7/53.3 (4-year CPFS/BPFS)
Schmidt-Hegemann [64]	2020	Retrospective analysis	5	135/135	16	PSMA-PET/CT, PSMA-PET/MRI	LN 38%, bone or viscera 62%	LN: 50.9 Gy, Bone 56 Gy, Viscera: 93.93 Gy (EQD2, α/β 1.5)	35%	NR	91% at last FU, median 16 month	46% at last FU, median 16 month
Phillips [13]	2020	Prospective randomized phased II study	3	54 (36 SBRT, 18 observation)/73	19	CT, MRT, Bone Scan	LN only 58%, bone-involved 42%	19.5–48 Gy/3–5 fr	0%	NR	98.9% (6-mo)	19 vs 61% (6-mo PFS), 11 vs 50% (6-mo BPFS)
Oehus [25]	2020	Retrospective analysis	5 bone or viscera, no limit for LN	78/185	16	PSMA-PET/CT	LN 69.2%, bone 24.3%, 6.5% viscera	LN: 50.9 Gy, Bone 51.4 Gy, Viscera: 64.7 Gy (EQD2, α/β 1.5)	16.7%	34	NR	55% 1-year bRFS
Koerber [72]	2020	Retrospective analysis	5	86/168	26	PSMA-PET/CT	LN 63%, bone 37%	SBRT: 27–37,5/3–5 fr, 61.2 Gy/34fr	61.9	13.5	90.9% at median 13 mo	85/55% (2/3 year BRFS)
Hurmuz [11]	2020	Retrospective analysis	5	176/353	23	PSMA-PET/CT	LN 43%, bone 35%, both 23%	27 Gy (15–40 Gy)/1–5 fr, 60 (40–78) Gy/25–39 fr	79.5%	NR	93.2% (2-year)	63.1% (2 year BPFS)
Devos [58]	2020	Retrospective analysis	5	191/350	45	CT, Bone Scan, Cholin-PET/CT, PSMA-PET/CT	LN 71%, bone 24%, viscera 6%	30 Gy/3 fr or 62.5/25 fr	61%	66 month	NR	median BRFS 8 month, median CPFS: 30 month
Reverberi [65]	2020	Retrospective analysis	5	37/NR	56	MRI, Cholin-PET/CT	LN 57%, bone 51%, both 19%	45–60 Gy/25 fr, SBRT dose NR/1–5 fr	97.3%	NR	93.9/83.7% (2/5 year)	79.1/55.4% (2/5 year CPFS) 73.3./39.3% (2/5 year BPFS)
Kalinauskaite [12]	2020	Retrospective analysis	5	50/75	34	PSMA-PET/CT	LN 48%, bone 46%, both 4%, viscera 2%	16–28.8 Gy/1–3 fr	30%	2 year ADT-FS: 60.5%	98/96% (1/2 year)	54/22% (1/2 year BPFS)
Deek [27]	2019	Retrospective analysis	5	156/354	25	CT, MRT, Choline-PET/CT, PSMA-PET/CT, NaF-PET/CT, Fluciclovine-PET/CT,	LN 43%, bone 52%, visceral 3%	15–20 Gy/1 fr; 24–36 Gy/3 fr, 30–50 Gy/5 fr	59.6%	27.8	96.1%/93.6% (1/2 year)	52% 1-year BRFS
Deek [44]	2020	Retrospective analysis	5	258/474	25	CT, MRT, Choline-PET/CT, PSMA-PET/CT, NaF-PET/CT, Fluciclovine-PET/CT,	LN 28%, bone 62%, both 10%	15–20 Gy/1 fr; 24–36 Gy/3 fr, 30–50 Gy/5 fr	50.4%	NR	96/93% (1/2 year)	median BPFS 16 month, median DMFS 19 month

*BPFS* biochemical progression-free survival, *CFFS* clinical failure-free survival, *CPFS* clinical progression-free survival, *CRFS* clinical relapse-free survival, *CT* computed tomography, *DPFS* distant progression-free survival, *ENRT* elective nodal radiation therapy, *FDG* Fluordesoxyglucose, *FFDP* freedom from distant progression, *FFR* failure-free rate, *FU* follow-up, *LN* lymph node, *MFS* metastasis-free survival, *MRI* magnetic resonance imaging, *NaF* sodium fluorid, *NR* not reported, *OR* oligoreccurent, *OP* oligoprogressive, *PSMA* prostate-specific membrane antigen, *SBRT* stereotactic body radiation therapy, *SD* single dose, *TD* total dose

Whether the number of metastases also has prognostic value within the collective of oligometastatic patients remains unclear. While some studies - possibly underpowered due to small patient cohorts - could not show any influence, the number of metastases had an impact on the outcome in other studies [6–12].

Data for local control (LC) and progression free survival (PFS) are shown in Table 1. LC rates ranged between 76 and 100% at 2 years. PFS was inconsistently defined, as biochemical progression, clinical progression or both. The reported PFS values ​​ranged from 38 to 100% at 1 year and 22–83% at 2 years and median PFS rates ranged from 7 to 63 month. The ORIOLE Trial, (a randomized phase II study) compared observation and MDT, and showed a significant difference in the median PFS with MDT (not reached vs. 5.8 months; hazard ratio, 0.30) [13]. Due to the large number of small case series, patient collectives, therapies and predictive factors differed substantially. Hereinafter, some of these factors and their predictive value will be discussed in detail.

### Site of oligometastasis: bone versus lymph node

The sites of treated metastases in the studies were mostly bone or lymph node. In the present review, twelve, seven and 37 studies with treatment of exclusively nodal metastases, bone metastases or both were included and investigated. In most studies including patients with nodal and bone metastases, the site of metastasis was not a predictive factor for the respective clinical outcomes [10, 12, 14–25]. In contrast, Fodor et al. reported a higher risk for clinical relapse in patients with extra-pelvic lymph nodes metastases compared with pelvic lymph node lesions and in the studies of Schick et al. and Deek et al. a trend for better biochemical progression-free survival (BPFS) was shown in patients with lymph node metastases compared with those with bone metastases [6, 26, 27]. In addition, the largest study to date based on prospectively collected data based on patients treated on clinical trials, demonstrated that the presence of bone metastases was associated with a worse survival compared to lymph node metastases in MPC [28]. Hence, it is not surprising that in the recently published APCCC report, the majority of experts voted for the distinction of these two kinds of metastatic patterns [29]. However, since encouraging clinical outcomes of studies with exclusively bone metastases were reported, with 2-year LC and PFS rates of 76–100% and 27–38%, respectively, these patients may benefit from MDT and should not be excluded [8, 30–35].

### Imaging methods

Due to the lack of predictive biomarkers, the definition of oligometastasis is currently based on the sheer number of metastases as determined by imaging, underscoring the critical importance of reliable imaging. Staging with ^68^Ga-prostate-specific membrane antigen PET/CT (PSMA PET/CT) appears to show the highest detection rates of metastases compared to other imaging modalities till now [36]. High detection rates of 15–58%, 25–73% and 69–100% were reported for PSA ranges of 0.2–0.5 ng/ml, 0.5–1.0 ng/ml and 1–2 ng/ml, respectively [37–41]. Compared to Choline PET/CT, PSMA PET/CT is substantially more sensitive, especially for low PSA values less than 2 ng/ml [42, 43].

Therefore, due to the lower detection rates in studies that did not use PSMA PET/CT as imaging, many patients may have been yet undiagnosed polymetastatic disease and were consequently understaged [44]. In fact, even staging with PSMA PET/CT cannot exclude this possibility, but it can be assumed that this modality comes closest to defining a “true” oligometastatic state.

Two of the included studies investigated staging with choline or PSMA PET/CT as predictive factor in univariate analysis but failed to detect any impact of imaging on LC, PFS, OS or treatment escalation [19, 22]. However, small case numbers may limit the statistic power to prove a significant difference.

Despite the absence of definitive evidence for superiority of PSMA PET/CT in the oligometastatic setting, there has been a remarkable increase in use of ^68^Ga -PSMA PET/CT imaging in recent years. While 17% of the studies in this review published in 2017 used at least in part PSMA PET/CT, it was 47% and 78% of the studies in 2018 and 2019 [20–23, 32–34, 42, 45–55]. Being in line with these data, the panelists of APCCC recommended PSMA PET/CT to confirm the diagnosis of an oligometastatic disease after radical treatment [5]. A PSA threshold of 0.3 to 0.83 ng/ml appears to be an optimal cut-off value for using PSMA PET/CT as staging [50, 51, 54].

### Synchronous versus metachronous disease

As used in the literature, oligometastasis can be defined to be present if detected either synchronously at the time of diagnosis of the primary tumor or metachronously (at a later date). However, the former scenario was regarded by some experts simply as metastatic disease. Moreover, there is no consensus in literature on the exact interval between diagnosis of the primary tumor and detection of oligometastases to differentiate between metachronous versus occult synchronous disease. Nevertheless, a frequently used definition of metachronous disease is an interval of more than 6 months [56]. Although the vast majority of the studies included patients with recurrent, i.e. “metachronous” disease, the reported intervals between primary diagnosis and detection of metastases were often less than 6 months. These studies had therefore rather mixed populations with metachronous and synchronous metastatic disease.

The parameters “time between primary and detection of oligometastasis” or “time between primary and radiotherapy” were reported in 35 studies with a median time interval between 7 and 67 months (range 0–240 months). Only 13 studies evaluated and reported one of these parameters in univariate or regression analysis, nine of them found no impact on outcome [15, 19, 20, 22, 24, 26, 48, 49, 57, 58]. In contrast, Lépinoy et al. showed that a dichotomous division of patients by interval between primary and oligometastasis of more or less than 5 years was predictive for failure in both univariate and multivariate analyses with better outcome for intervals longer than 5 years [59]. Similarly, Ong et al. reported a better distant progression-free survival with longer time intervals and Kalinauskaite found an improved treatment failure free-survival in patients with time to first metastasis longer than 36 months [12, 23]. In accordance with this data, it seems rational that a longer interval between primary diagnosis and oligometastasis may indicate less aggressive tumor biology. Metachronous disease was also an inclusion criterion for the two major phase II studies STOMP and ORIOLE addressing MDT in metastatic prostate cancer [13, 60].

### Systemic therapies

Since it is widely accepted that hormone-sensitive prostate cancer (HSPC) and castration-resistant prostate cancer (CRPC) are different entities in terms of tumor biology and prognosis, it is consequential that in most studies the status of hormone sensitivity was reported [7, 12, 13, 17, 19, 22, 30, 32, 34, 35, 44, 49, 50, 53, 54, 58, 61–65]. In the study of Franzese et al. CRPC was an independent risk factor for inferior PFS compared to HSPC in multivariate analysis (HR 2.12, *p* = 0.021) [19]. This was confirmed by the data reported by Patel et al. (HR 8.43, *p* < 0.001) [34]. In addition, Guler and Deek reported a significant worse PFS in CRPC patients [27, 50]. The reasons could be the more aggressive tumor biology in CRPC or/and a worse response to MDT. In our opinion, HSPC and CRPC should be considered as two distinct subgroups for further studies of oligometastasis.

Little is reported about the influence of hormone sensitivity on LC rates. Deek et al. found a significant higher local failure rate in CRPC patients compared with HSPC patients and Franzese et al. confirmed CRPC as a predictive factor for worse LC in univariate analysis [19, 27]. However, this effect was no longer detectable in the multivariate analysis. LC rates in the mixed-group studies were similar to those in which only HSPC patients were included. The 2-year local control reported by Triggiani et al. was 92.8% and 90.2% for HSPC and CRPC, respectively, so that it can be concluded that SBRT was able to achieve an excellent LC rate in both CRPC and HSPC oligometastatic patients [18]. This is not surprising given the fact that most studies of RT palliation for bone metastases have reported high response rates [66].

The “standard of care” for MPC has been ADT alone until recently wherein combinations including other systemic agents such as abiraterone or docetaxel have been added [67–69]. Even more recently local irradiation of prostate has been to standard systemic treatment and shown to improve overall survival for men with de novo metastatic prostate cancer with low metastatic burden [70]. However, some patients refuse systemic treatment primarily due to fears concerning their potential side effects and their comorbidity. Thus, androgen deprivation therapy free survival (ADTFS) was introduced by some authors in HSPC patients and reported in several studies, which ranged between 7 and 66 months [9, 12, 14–18, 25, 27, 32, 46, 57, 58, 71, 72]. Similarly, in CRPC cohorts, systemic therapy-free survival and treatment escalation-free survival, ranging between 16 and 27 months, were also introduced in the management of prostate cancer and investigated in some studies [22, 24, 47]. In the case of newly developed oligometastasis after the initial metastasis-directed therapy, a second and further SBRT was allowed in some of these studies.

Of particular note is the prospective randomized STOMP study, which showed a prolonged ADTFS with MDT compared to observation after a medial follow-up of 3 years (21 vs. 13 month) [60]. LC and biochemical progression-free time were also improved in the MDT group with comparable quality of life. In the prospective single-arm trial reported by Siva et al., the ADTFS rate was 48% at 2 years. On the other side, there is a clear body of evidence showing improved overall survival with ADT and its combination therapies in metastatic disease [67, 73, 74]. Thus, omitting ADT may be associated with a worse survival while temporarily delaying side effects. This point should also be taken into account in decision-making of treatment and in the context of informed decision making with patients. Indeed 75% of the panelists of APCCC recommended adding MDT to systemic therapies, instead of replacing them [29].

### Radiation response, dose and field size

Baumann et al. examined the metabolic response rate in PSMA PET/CT after SBRT of bone metastases with 5 × 7 Gy [45]. 78% of the irradiated lesions showed a metabolic response, which correlated with the time interval between SBRT and the post-therapeutic PSMA PET/CT. The metabolic response rate was 100% when follow-up imaging was performed 5 months or longer after the radiation. Consequently, a time interval of at least 6 months was recommended for the post-therapeutic PSMA PET/CT as response evaluation.

The used fractionation schemes were highly variable (Table 1) ranging from single-dose SBRT with 24 Gy over total doses of 20–50 Gy (or more) in several fractions by moderately hypofractionated or normofractionated schedules. The most common fractionation scheme was 30 Gy in three fractions. Although in general the LC rate was high with acceptable toxicities, the optimal fractionation scheme remains undefined.

Some studies fail to show that radiation dose is predictive of outcome [17, 21, 24–26, 33, 53, 75], however, Ost et al. found better local PFS in multivariate analysis with a biological effective dose (BED) > 100 Gy, using an α/β value of 3 Gy [16]. This cut-off dose was supported by another study in which a BED > 100 Gy resulted in prolonged systemic treatment-free survival in univariate analysis [24]. In addition, Hurmuz and colleagues showed a better progression free-survival with a BED > 108 Gy [11]. Muldermans et al. reported a higher 2-year LC rate for SBRT with ≥ 18 Gy compared to 16 Gy (95% vs. 58%, *p* = 0.001). In another study of 40 patients, a median single-fraction dose of 20 Gy was used. Local failure occurred only in two patients who were treated with a reduced SBRT dose, because of prior radiotherapy and/or vicinity to dose-sensitive organs related at risk [7, 31]. Additionally, Schick et al. found a significantly improved BPFS for SBRT with EQD_2_ (equivalent dose in 2 Gy fractions) > 64 Gy using an α/β-value of 2 Gy (HR 0.37, *p* = 0.034) [6]. Although these observations may be considered “first hints” for defining the optimal dose, which should be taken into-account in the designing of further clinical trials, caution must be exercised in assuming that these post hoc studies are definitive due to issues related to major patient selection biases.

Regarding MDT of lymph node metastases, a distinction must be made between SBRT of the affected lymph nodes only and prophylactic elective nodal radiation therapy (ENRT) of the (loco)-regional lymph node station. ENRT usually involves using conventionally fractionated (i.e. 1.8–2.0 Gy) to imaging negative nodes to 45–50 Gy with a boost to the affected (i.e. PET positive) lymph nodes [6, 26, 76]. SBRT of lymph node metastasis was performed in a single fraction or hypofractionated with doses between 24 and 50 Gy in 3–10 fractions. Some studies reported a type of “involved field” irradiation without inclusion of the whole ipsilateral lymphatic drainage [21, 51, 54]. The doses used were 45–60 Gy with a boost up to total doses ranging from 63 to 74 Gy.

In two studies, the authors directly compared SBRT to ENRT plus Boost: Lépinoy et al. compared SBRT of affected lymph nodes mostly using 36 Gy in 5 fractions to conventionally fractionation extended field irradiation of the whole pelvis [59]. The use of ENRT was associated with a significantly longer failure-free time, albeit with a little more acute gastrointestinal toxicity. Their results were confirmed by De Bleser et al., who also reported fewer nodal recurrences and higher late toxicity in the ENRT group [48]. These findings and the pattern of progression described below support the hypothesis that in some cases, despite improved imaging sensitivity, the extent of metastasis, especially the spreading of microscopic cancer cells, is underestimated.

### Pattern of progression

Distant/regional progression-free survival after MDT was 27–45% after 2 years [15, 51, 54, 62, 63]. Of the patients, who relapsed after the initial MDT, 50–91% relapsed again in an oligometastatic pattern (as defined in the initial definition of oligometastasis in each study) [15, 54]. A second, third and fourth course of SBRT was administered in some studies without increased toxicity [12, 15, 51, 54, 58, 62, 63, 65]. In the trials using SBRT for MDT, recurrences occurred mostly in the same organ system, in lymph nodes or bone, respectively [15, 17, 44]. Moreover, Nicosia et al. described that the majority of patients with nodal recurrence after SBRT suffered a lymph node relapse, which was out of but close to the radiation fields [53]. Soldatov et al. reported a shift from iliac lymph node metastases to retroperitoneal lymph node metastases or from retroperitoneal to distant lymph node metastases and bone metastases in patients with oligometastatic lymph nodes treated with ENRT [54]. This might be explained by the coverage of adjacent lymph nodes or elective lymph node stations. Moreover, the radiation dose for elective lymph node stations in the ENRT approach seems to be sufficient to eliminate the microscopic tumor cells, in principle favoring extended irradiation fields in this regard. However, less toxicities and the feasibility of repeated radiotherapy and possibly an enhanced immune response as shown in the ORIOLE trial potentially supporting the rationale for the use of SBRT alone [13].

## Conclusion and future perspectives

The present review summarizes the available evidence on MDT in patients with what is commonly called “oligometastatic” prostate cancer. Unfortunately, there is a lack of consistency as to how “oligometastatic” disease is defined how it was treated, and the endpoints used to assess outcomes. In addition, due to rapidly evolving nature of imaging, the complexities involved in determining optimal management of oligometastatic prostate cancer diseases cannot be resolved today. Nonetheless, low morbidity and high local control rates have been reported with a considerable proportion of patients (22–83%) remained progression-free for 2 years. With its relatively high sensitivity and specificity (compared to other imaging approaches), PSMA PET/CT was increasingly used for staging and for defining this entity. Although to date there is no randomized data demonstrating a better clinical outcome by using PSMA PET/CT for oligometastatic disease, it can be assumed that the higher detection rate will allow more patients to be diagnosed earlier in the metastatic course. This is supported by the multicenter retrospective cohort study of Mazzola and colleagues, in which PSMA-PET-guided SBRT for oligorecurrent castration-sensitive PC lead to a higher rate of ADT-free patients when compared with the 18F-choline-PET cohort [77].

Till now, the definition of oligometastasis was based on the sheer number of metastases, without taking into account the inhomogeneous biologic characters of cancer diseases and the potentially critical distinction between synchronous and metachronous metastasis. This may explain the inconsistencies in the results reported in different studies. The limitations associated with a definition based solely by the number of “oligometastasis” and on imperfect imaging, means that we are doomed to have an imperfect definition. Other risk factors, such as Gleason score, PSA kinetics, should also be involved in the differentiation of the oligometastatic diseases in the future. Moreover, given its rapid evolvement in the last years and great potential for precise risk stratification, novel biomarkers may be helpful for identifying patients who benefit from MDT.

Treatment regimens varied widely in radiation dose and field size. A possible cut-off value of radiation dose could be considered at BED > 100 Gy. In lymph node irradiation, a more extensive ENRT seemed to be superior to SBRT in terms of loco-regional disease control, albeit at cost of slightly higher incidence of acute toxicity. With the recent completed enrollment of > 2500 patients on to RTOG 0924 trial (evaluating the impact of prophylactic nodal irradiation) and a planned analysis in 2023), the understanding and management of micro-metastatic disease (possibly below the resolution of PSMA-PET) is likely to change.

Although, the role of ADT in the oligometastatic patients treated with MDT remains an unsolved issue, it seems most highly implausible that RT alone will ever be adequate. While there is evidence from a phase II study for a prolonged ADTFS with MDT in oligometastatic patients, concurrent ADT seemed to improve the effectivity of MDT in some other retrospective series. Thus, future studies should be designed to clarify the role of ADT in oligometastatic diseases, especially in the context of the widespread usage of MDT. It may be possible that different subgroup of oligometastatic patients benefit from different therapy approach, which also need to be addressed. Several prospective studies on MDT in oligometastatic prostate cancer are ongoing [36, 78–81]. Their final results will hopefully provide more solid evidence for the optimal usage of MDT in clinical practice.

## Data Availability

Research data are stored in an institutional repository and will be shared upon request to the corresponding author.

## Abbreviations

ADT: Androgen deprivation therapy
ADTFS: Androgen deprivation therapy-free survival
APCCC: Advanced prostate cancer consensus conference
BPFS: Biochemical progression-free survival
BED: Biologically effective dose
CT: Computed tomography
CRPC: Castrate-resistant prostate cancer
ENRT: Elective nodal radiotherapy
HSPC: Hormone-sensitive prostate cancer
LC: Local control
MDT: Metastasis-directed therapy
MPC: Metastatic prostate cancer
PET: Positron emission tomography
PET/CT: Positron emission tomography/computed tomography
PRISMA: Preferred reporting items for systematic reviews and meta-analysis
PFS: Progression-free survival
^68^ Ga-PSMA: ^68^Gallium-prostate-specific membrane antigen
PC: Prostate cancer
PSA: Prostate specific antigen
RT: Radiotherapy
SBRT: Stereotactic body radiation therapy
